# Antiseptic Surgery

**Published:** 1890-03

**Authors:** W. F. Westmoreland

**Affiliations:** Lecturer on Fractures, Dislocations and Antiseptic Surgery, in the Atlanta Medical College, Atlanta, Ga.


### ANTISEPTIC SURGERY.

BY W. F. WESTMORELAND, JR., M. D., LECTURER ON FRACTURES, DISLOCATIONS AND ANTISEPTIC SURGERY, IN THE ATLANTA MEDICAL COLLEGE, ATLANTA, GA.—[CONCLUDED.]

In continuation of an article on antiseptic surgery that appeared in the January number of this Journal, we will speak of the preparation of antiseptic materials.

### GAUZES.

Gauzes which are prepared by houses dealing in antiseptic materials are cheaper, in the long run, and generally much more satisfactory in their action, although with the use of some little care the surgeon can prepare a very reliable article at home. For the first year that I used antiseptic surgery I prepared my own gauze, and with it I had good results invariably.

The main consideration in preparing a gauze is to make it with the highest amount of absorptive power possible. To accomplish this, you wish a very light, wide mesh goods. What is known in the commercial world as a light cheese cloth is the best goods, and one that you can generally find at any store. Be certain and get one with a wide mesh.

This goods, as it comes from the store, will absorb about three and a half times its weight of moisturé. This is not sufficient, and can be increased, as follows: I cut the goods into five yards strips, so as to handle it very easily, then put it into a strong solution of carbonate of soda, which is kept in any drug store, and let it remain for a few hours. This strong alkaline solution will remove the oil and extractive matter from the cloth and increases its power of absorption to about five times its weight of moisture, which is a considerable gain. Every trace of the alkaline solution must be removed by repeated washings in clear water. Never use boiling water, as it will cause the fibres of the cloth to contract, on account of the heat, and destroys the power of absorption to that extent. When the alkali is thoroughly removed, hang up the gauze in a clean room, where it will not be exposed to dust and dirt, to dry.

After it is thoroughly dried, put it in the following solution: Four hundred and ninety parts of water (490), ten parts o glycerine (10), and one part of bi-chloride of mercury. Let it remain in this solution for several hours.

This solution is really one to five hundred (1-500) bi chioride sol., with the ten parts of glycerine added. The object of the glycerine is to more thoroughly hold the solution of mercury in the meshes of the gauze and to prevent it from evaporating as rapidly as it otherwise would. The glycerine also has a powerful affinity for moisture, and by the possession of this property, increases the power of absorption by the gauze. When the gauze is thoroughly impregnated with the solution, wring it out and hang it up to dry. When dry, place either in glass jars or a wooden box. This gauze is now ready to use.

In using the gauze prepared in this way, it was always my custom, just before using it as a dressing, to place it in the solution (1-500 bi. chl. sol. with 10 parts glycerine added) mentioned above, and when ready to use it, wring it out thoroughly and use moist. I was then always certain that I got more thorough antisepsis and also have the superior advantage of the greater absorption power of the moist over the dry dressing, the discharge from the wound percolating more thoroughly and rapidly through the moist than through the dry dressing.

Iodoform gauze can be made by taking the gauze, as prepared above, and sprinkling the iodoform on it and rubbing it thoroughly and evenly into its meshes.

ABSORBENT COTTONS.

The absorbent cottons, which we use as other dressings, are of two kinds; the borated cotton, which is used next to the gauze, and the plain absorbent cotton, which is used over the borated cotton. Borated cotton is a plain absorbent cotton, thoroughly impregnated with boracic acid. The plain absorbent cotton is prepared by removing all the oil and extractive matter from the plain cotton.

The cotton had best be bought ready prepared, as you can not make them satisfactorily.

DRAINAGE.

Drainage can be made by using either cat-gut strands, bone or rubber drainage tubes.

The use of cat-gut strands for drainage is limited to small superficial wounds, where only a small amount of drainage is needed. Temporarily, that is, for a day or two, it succeeds very well in these cases. The cat-gut used is the same as is used for sutures. A few strands, their size depending upon circumstances, determined in each particular case, are placed in the most dependent angle of the wound. The preparation of the cat-gut will be gone into more particularly under the head of sutures.

Bone drainage tubes had best be bought already prepared, as their preparation is very difficult. They can be used in such wounds as amputation of breast, and operations of that class, where it is only necessary to keep up drainage for about a week. The great advantage of using catgut or bone drainage tubes is that they become absorbed and the dressing does not have to be taken off to remove them, as it does when rubber drainage tubes are used.

RUBBER DRAINAGE TUBES I generally prepare myself. I get the ordinary black rubber tubing of the various sizes I may need. It is generally kept at any drug store or place handling rubber goods. I always get the unbleached black rubber tubing, and not the white tubing which has been bleached; sulphur is used in bleaching it, and not only makes it more brittle and hard, but when it is used around a wound bleaches the whole surface that it comes in contact with, and gives it a sulphurous smell that is decidedly unpleasant.

The rubber drainage tubes are used in such wounds as require permanent drainage—that is, where drainage is necessary for from one week to several months. There are two methods of removing these drainage tubes—first, where there is a recent wound, as in an amputation of the leg, and you remove the tube entirely at the first dressing; and secondly, where you cut off a short piece of the tube at each dressing, making it shorter each time, as the indications may necessitate it, as in abscesses, etc.

### SUTURES.

Sutures that are generally used are of three kinds—silver wire, silk and catgut. The first two are of course bought ready prepared, and are easily kept antiseptic; the first, silver wire, in whatever sizes are necessary, are kept in carbolized oil (1-10), that is, one part of carbolic acid to ten parts of sweet oil. It is rarely ever necessary to use wire in any operation that is done antiseptically.

SILK SUTURES in whatever sizes necessary can be kept in an aqueous sol. carbolic acid (1-20). They are rarely used now, except in special operations, as hair lips, etc.

CATGUT SUTURES are the most frequently required in antiseptic operations. Their greatest value lies in the fact that whether they are used as sutures or ligatures they are completely absorbed, the rapidity of the absorption depending entirely on the size of the strand, which is animal tissue.

I always prepare my own catgut sutures. I buy the commercial catgut from the drug store, wash it thoroughly with a nail brush and castile soap, afterwards rinsing the soap suds from it with clean water. The catgut is then placed in a solution of bi-chlor. mer. (1-1000) for twelve to twenty-four hours, the length of time depending on the size of the gut. Do not allow it to remain in this solution longer than this, or it will make it too soft and soddy for use, and this is plenty long to thoroughly disinfect it. When taken from the bi-chlor. sol. put it in alcohol, where it is kept permanently. I have found the commercial alcohol of sufficient strength to preserve it if renewed occasionally as it evaporates.

SPONGES.

I prepare my sponges in the following manner: I generally buy them by the pound. Put a gallon of water into a bucket and add eight ounces of a saturated solution of permanganate of potassium; put in all the sponges that the solution will cover; let them remain until they become a nut brown color, then take them out and wring as dry as possible. To a gallon of water in another bucket add three fluid ounces of a concentrated solution hydrochloric acid and one ounce of hyposulphite of soda; put in a few sponges at a time and let them remain until they turn white. When they fail to turn promptly, add more of the acid and soda.

When the sponges are bleached squeeze out all the water and place in a dry, shady place. When they are perfectly dry have the sand beaten out and place them in a solution of carbolic acid (1-20), and in a day or two they are ready for use.

I find a large-mouth fruit jar the most convenient thing to keep them in.

SOLUTIONS.

It is not within the scope of this paper to enter into a discussion of the merits or demerits of the numerous antiseptic agents that have been proposed. I think that the bi-chloride of mercury is decidedly the best.

In making up solutions, sixteen grains of bi-chlor. mer. to one quart of water, in round numbers, make a one to one thousand solution (1-1000).

The tablets which are prepared contain seven and seven-tenths of a grain of mer., and one of them to a quart of water gives the bi-chloride sol. (1-2000), which is the strength generally used. These tablets are very convenient, they can be easily carried, and are much more easily dissolved than the bichlor. mer. itself, which is very insoluble. If the tablets are not easily procurable, you can prepare your own solution. Take an eight-ounce bottle (or any other size that you desire) and fill with water, add sixteen grains of mercury for each ounce of water in the bottle, and dissolve it thoroughly; use warm water if necessary. Half an ounce of this solution to a quart of water will give (1-2000) bi-chloride solution.

The bi-chlor. sol. is not only used for irrigating the wound, but also to place towels, sponges, etc., in, and for washing hands, during operation.

Carbolized solutions are only used to keep materials in, as already mentioned, and as a solution to put instruments in at time of operation. The strength of the solution for this purpose is one to forty (1-40).

---

The memorial window to the late Prof. James L. Cabell, at the University of Virginia, is being prepared, and will bear an inscription chiefiy indicative of his relationship to the University. We wish it could bear the record of many of his other important noble works, so as to perpetuate, in gilded letters, for coming generations, his history, so as to show that he belonged to the State, to the Nation—to humanity at large no less than to the University of Virginia.

